# Supplementary material for: A Scoping Review of Infrared Spectroscopy and Machine Learning Methods for Head and Neck Precancer and Cancer Diagnosis and Prognosis
Source: Cancers (Basel). 2025 Feb 26;17(5):796. doi: 10.3390/cancers17050796 (PMC11899414; doi:10.3390/cancers17050796)
Supplement: Supplementary file 1 [file cancers-17-00796-s001.zip › cancers-3457561-supplementary.pdf]

Table S1. Search strategy and keywords

Database	Keywords	Notes
MEDLINE	1. exp Artificial Intelligence/ or exp neural networks, computer/ or Fuzzy Logic/ or Random Forest/ or Least Square Analysis/ or Discriminant Analysis/ or Clustering Analysis/ or (machine-learning or transfer-learning or deep-learning or hierarchical-learning or hierarchical-clustering or artificial-intelligence or artificial-neural-network$ or convolutional-neural-network$ or classification-algorithm or feature-detection or feature-extraction or cross-validation or fuzzy-system$ or K-nearest-neighbor or outlier-detection or random-forest or support-vector-machine or partial-least-squares-regression or principal-component-regression or least-square-analysis or discriminant-analysis or clustering-algorithm).ti,ab,kw.<br>2. exp Spectrophotometry, Infrared/ or Hyperspectral Imaging/ or (Fourier-transform-infrared-spectroscopy or FTIR or hyperspectral-imag$3 or infrared-spectroscopy or Infra-red-spectroscopy or ir-spectra$1 or ir-spectroscopy or vibrational spectroscopy or vibration-spectroscopy).ti,ab,kw.<br>3. exp Neoplasms/ or exp Neoplasms by Histologic Type/ or exp Precancerous Conditions/ or (cancer or cancerous or carcinoma$ or leukemia$ or sarcoma$ or precancer$ or premalignan$ or preneoplastic or malignancy or malignant or neoplasm$1 or tumor$1 or tumour$1 or dysplasia$).ti,ab.<br>4. 1 and 2 and 3<br>5. limit 4 to english language<br>6. limit 5 to journal article	Ahead of Print, In-Process, In-Data-Review & Other Non-Indexed Citations and Daily <1946 to January 12, 2024>
Embase	1. 'artificial intelligence'/de OR 'machine learning'/de OR 'deep learning'/de OR 'artificial neural network'/de OR 'convolutional neural network'/de OR 'classification algorithm'/exp OR 'cross validation'/exp OR 'feature detection'/de OR 'feature extraction'/de OR 'fuzzy system'/de OR 'K nearest neighbor'/de OR 'outlier detection'/de OR 'supervised machine learning'/de OR 'Random forest'/de OR 'support vector machine'/exp OR 'supervised machine learning'/de OR 'unsupervised machine learning'/de OR 'partial least squares regression'/de OR 'principal component regression'/de OR 'least square analysis'/de OR 'discriminant analysis'/de OR 'clustering algorithm'/exp OR 'machine-learning':ti,ab,kw OR 'transfer-learning':ti,ab,kw OR 'deep-learning':ti,ab,kw OR 'hierarchical-learning':ti,ab,kw OR 'hierarchical-clustering':ti,ab,kw OR 'artifical-intelligence':ti,ab,kw OR 'artificial-neural-network':ti,ab,kw OR 'convolutional-neural-network':ti,ab,kw OR 'classification algorithm':ti,ab,kw OR 'feature-detection':ti,ab,kw OR 'feature-extraction':ti,ab,kw OR 'cross-validation':ti,ab,kw OR 'fuzzy-system*':ti,ab,kw OR 'K-nearest-neighbor':ti,ab,kw OR 'outlier-detection':ti,ab,kw OR 'random-forest':ti,ab,kw OR 'support-vector – machine':ti,ab,kw OR 'partial-least-squares-regression':ti,ab,kw OR 'principal-component-regression':ti,ab,kw OR 'least-square-analysis':ti,ab,kw OR 'discriminant-analysis':ti,ab,kw OR 'clustering-algorithm':ti,ab,kw<br>2. 'infrared spectroscopy'/de OR 'fourier transform infrared spectroscopy'/exp OR 'hyperspectral imaging'/de OR 'vibrational spectroscopy'/de OR 'fourier-transform-infrared-spectroscopy':ti,ab,kw OR 'ftir':ti,ab,kw OR 'hyperspectral-imag*':ti,ab,kw OR 'infrared-spectroscopy':ti,ab,kw OR 'Infra-red spectroscopy':ti,ab,kw OR 'ir-spectra*':ti,ab,kw OR 'ir spectroscopy':ti,ab,kw OR 'vibrational spectroscopy':ti,ab,kw OR 'vibration spectroscopy':ti,ab,kw	

	3.'malignant neoplasm'/exp OR 'carcinoma'/exp OR 'leukemia'/exp OR 'precancer'/exp OR 'neoplasms by histologic type'/exp OR cancer:ti,ab,kw OR cancerous:ti,ab,kw OR carcinoma*:ti,ab,kw OR leukemia*:ti,ab,kw OR sarcoma*:ti,ab,kw OR precancer*:ti,ab,kw OR premalignant*:ti,ab,kw OR preneoplastic:ti,ab,kw OR malignancy:ti,ab,kw OR malignant:ti,ab,kw OR neoplasm*:ti,ab,kw OR tumor*:ti,ab,kw OR tumour*:ti,ab,kw OR dysplasia*:ti,ab,kw<br>4. #1 AND #2 AND #3<br>5. #4 AND 'article'/it<br>6. #4 AND 'article'/it AND [english]/lim	
Scopus	(TITLE-ABS-KEY ("machine learning" OR "transfer learning" OR "deep learning" OR "hierarchical learning" OR "hierarchical clustering" OR "artificial intelligence" OR "artificial neural network*" OR "convolutional neural network*" OR "classification algorithm" OR "feature detection" OR "feature extraction" OR "cross validation" OR "fuzzy system*" OR "k nearest neighbor" OR "outlier detection" OR "random forest" OR "support vector machine" OR "partial least squares regression" OR "principal component regression" OR "least square analysis" OR "discriminant analysis" OR "clustering algorithm")) **AND** (TITLE-ABS-KEY ("fourier transform infrared spectroscopy" OR "ftir" OR "hyperspectral imag*" OR "infrared spectroscopy" OR "infra red spectroscopy" OR "ir spectra*" OR "ir spectroscopy" OR "vibrational spectroscopy" OR "vibration spectroscopy")) **AND** (TITLE-ABS-KEY (cancer OR cancerous OR carcinoma* OR leukemia* OR sarcoma* OR precancer* OR premalignant* OR preneoplastic OR malignancy OR malignant OR neoplasm* OR tumor* OR tumour* OR dysplasia*)) AND (LIMIT-TO (DOCTYPE , "ar")) AND (LIMIT-TO (LANGUAGE , "english"))	
